# Plant Monoterpenes Geraniol, Eugenol and Carvacrol Against Multidrug-Resistant ESKAPE Isolates from Surgical Wounds

**DOI:** 10.3390/antibiotics15090869

**Published:** 2026-09-06

**Authors:** Marija Radovanović, Stanislava Čukić, Jelena Filipović Tričković, Jadranka Miletić Vukajlović, Biljana Nikolić, Jelena Marinković

**Affiliations:** 1 Department of Physical Chemistry, “Vinča” Institute of Nuclear Sciences, National Institute of the Republic of Serbia, University of Belgrade, Mike Petrovića Alasa 12, 11351 Belgrade, Serbia; 2 Department of Laboratory Diagnostics, University Hospital Center “Dr Dragiša Mišović-Dedinje”, Heroja Milana Tepića 1, 11000 Belgrade, Serbia; 3 Department of Microbiology, Faculty of Biology, University of Belgrade, Studentski trg 16, 11158 Belgrade, Serbia

**Keywords:** antibiofilm effect, binary mixtures, cytotoxicity intrahospital pathogens, minimal inhibitory and bactericidal concentrations

## Abstract

**Objectives:** The study determined antimicrobial resistance profiles of surgical wound multidrug-resistant (MDR) isolates belonging to the ESKAPE group and evaluated antibacterial and antibiofilm activities of geraniol (G), carvacrol (C) and eugenol (E), individually and in the selected mixtures. The cytotoxicity of monoterpenes and combinations was also assessed. **Methods:** The antibacterial and antibiofilm activity against tested isolates of *Enterococcus faecium*, *Staphylococcus aureus* MRSA, *Klebsiella pneumoniae*, *Acinetobacter baumannii*, *Pseudomonas aeruginosa* and *Enterobacter* sp. was assessed in microdilution and crystal violet assay, respectively, while cytotoxicity was estimated in XTT assay on human MRC-5 fibroblasts. **Results:** C showed the strongest antibacterial activity (MIC 0.32 ± 0.24 mg mL^−1^). Monoterpenes induced synergism in certain combinations (FICI 0.09–0.31). All individual monoterpenes inhibited biofilm formation, but G was the most active (38.78–84.72%, *p* < 0.05). The most pronounced biofilm eradication was observed for C (25.21–61.34%, *p* < 0.05). G-C mixtures showed notable inhibition of biofilm formation in all isolates except *P. aeruginosa*, but, on the contrary, induced biofilm eradication against *P. aeruginosa* only. Cytotoxicity was not detected with the applied concentrations of monoterpenes, while the G-C mixtures exhibited lower cytotoxicity than povidone-iodine (*p* < 0.05) used as the control. **Conclusions:** All monoterpenes and G-C mixtures proved significant antibacterial and antibiofilm potential against ESKAPE isolates and acceptable impact on cell viability. **Practical significance:** The results highlight the potential of plant monoterpenes and their binary combinations as complementary agents for controlling MDR ESKAPE pathogens and biofilm-associated infections following surgical procedures. Further in vivo and safety studies are needed to confirm their applicability in clinical settings.

## 1. Introduction

In 2017, WHO released, for the first time, a list of bacterial pathogens presenting a serious threat to human health, which is updated constantly [1,2]. Among other pathogens, the subgroup marked with the ESKAPE acronym contains the ones with “priority status”, namely *Enterococcus faecium*, *Staphylococcus aureus*, *Klebsiella pneumoniae*, *Acinetobacter baumannii*, *Pseudomonas aeruginosa* and *Enterobacter* spp. All specified are commonly isolated as multidrug-resistant pathogens (MDR), i.e., pathogens resistant to at least one agent belonging to three or more antibiotic classes [3]. Furthermore, subclasters of MDR pathogens were described in order to better estimate antimicrobial resistance (AR) grade: (1) extensively drug-resistant bacteria (XDR) including the strains featured with nonsusceptibility to almost all tested agents belonging to different antimicrobial classes, except only one or two agents belonging to the same or different categories and (2) pandrug-resistant bacteria (PDR) involving nonsusceptible strains to all tested agents belonging to all tested antimicrobial categories [4].

It has been suggested that the increased prevalence of MDR pathogens is driven by natural bacterial evolutionary mechanisms, genetic mutability and horizontal gene transfer, with the improper use and overuse of antibiotics further contributing to their emergence and spread, resulting in a phenomenon whereby infections previously regarded as routine are now becoming a serious threat [5]. Accordingly, surgical site infections (SSIs) are considered to be one of the most prevalent causes of hospital infections. In 70% to 95% of cases, constituents of the endogenous patient’s microbiome are causative agents of SSIs. It is commonly suggested that *Escherichia coli* and *Enterococcus* species contribute to 9.5% and 5.1% of all SSIs, respectively [6]. Moreover, *S. aureus* accounts for 24% of surgical site infections, and among them, methicillin-resistant *S. aureus* (MRSA) is associated with poorer clinical outcomes and extended hospital stay [6]. Further, Gram-negative ESKAPE pathogens, including genera *K. pneumoniae*, *P. aeruginosa*, *A. baumannii* and *Enterobacter* sp., have also been frequently identified among SSI pathogens, accounting for 12.27%, 8.80%, 0.6% and 5.60% of reported SSI pathogens, and mortality resulting from these infections has also been documented [7,8].

Taking into account aforementioned, usage of antiseptics is crucial for the prevention of infections in the postoperative period. The most commonly used povidone-iodine can inhibit fibroblast proliferation and cellular viability even in diluted forms, therefore negatively affecting wound healing [9]. Moreover, its frequent utilization could be associated with various health problems, such as acute renal failure, seizures and hyperchloremic acidosis, thus suggesting a need for alternative but efficient antimicrobial agents [9].

Promising alternatives could be seen in essential oils (EOs) and their constituents, since they are embedded with strong antibacterial activity and especially antibiofilm properties [10]. This investigation has focused on selected monoterpenoid constituents of EOs, namely geraniol (G), carvacrol (C) and eugenol (E), due to their strong and proven antibacterial potential. Selected monoterpenes are constituents of numerous EOs, but their high contents are typical for *Monarda fistulosa*, *Cymbopogon* spp. and *Pelargonium graveolens* in the case of G [11] *Origanum*, *Thymus*, *Coridothymus*, *Thymbra*, *Satureja* and *Lippia* genera in the case of C [12] and clove and betel pepper in the case of E [13].

Although G, C and E have previously been demonstrated to exhibit antibacterial activity against various bacterial species [14], including some members of the ESKAPE group, information regarding their efficacy against MDR ESKAPE strains remains limited. To the best of our knowledge, this study is the first to evaluate the antibacterial activity of G against MDR *E. faecium* and *P. aeruginosa*, as well as the activity of E and C against MDR *Enterobacter* spp. Similarly, data on their antibiofilm activity against multidrug-resistant ESKAPE pathogens are limited. In particular, this study provides the first evaluation of the antibiofilm activity of (1) G against *K. pneumoniae*, *P. aeruginosa*, *E. faecium* and *Enterobacter* sp.; (2) C against *E. faecium* and *Enterobacter* sp.; and (3) E against *E. faecium* and *Enterobacter* sp., all of which were MDR strains. Moreover, to the best of our knowledge, no published data are available on the antibiofilm activity of binary mixtures of these monoterpenes, prepared at clearly defined ratios, against biofilms formed by MDR ESKAPE strains.

Taking this into account, the aims of the study were (1) to collect surgical wounds isolates of the ESKAPE group, determine their antimicrobial resistance pattern and classify them as MDR, XDR or PDR, (2) to evaluate the antibacterial and antibiofilm potential of G, C and E applied individually and in the binary mixtures, against selected isolates belonging to ESKAPE, and (3) to screen for the cytotoxicity of the monoterpenes and their selected binary mixtures in order to provide preliminary risk assessment.

## 2. Results

Screening of the antibiotic resistance pattern pointed out that all tested isolates belonging to the ESKAPE group were MDR. However, *A. baumannii*, *P. aeruginosa* and *K. pneumoniae* belong to the XDR subgroup, while the *Enterobacter* sp. isolate could be denoted as PDR (Table 1).

### 2.1. Antibacterial Potential of Monoterpenes and Their Interactions

Comparison of the inhibitory potential among monoterpenes (Table 2) revealed that C (average MIC value 0.32 ± 0.24 mg mL^−1^) has achieved higher potential in comparison to E and G (*p* = 0.00544 and *p* = 0.02912, respectively). The activities of E and G were notable (MICs 2.69 ± 2.10 and 3.63 ± 3.08 mg mL^−1^, respectively) and similar (*p* = 0.340749). In addition, the inhibitory potential of all tested monoterpenes was significantly higher (*p* < 0.00001) in comparison to the positive control (povidone-iodine MIC 41.67 ± 16.70 mg mL^−1^).

Concerning strain sensitivity, it can be suggested that C seemed to be similarly efficient against all tested isolates. For both E and G, *A. baumannii* and *P. aeruginosa* were the most sensitive isolates. The least sensitive isolates were *E. faecium*, *S. aureus* MRSA and *K. pneumoniae* for E, and *E. faecium*, *K. pneumoniae* and *Enterobacter* sp. for G. Bactericidal effect of C was achieved on the lower tested concentrations (range 0.26 ± 0.00–1.06 ± 0.00 mg mL^−1^) in comparison to two other tested monoterpenes (0.68 ± 0.00–13.5 ± 0.00 mg mL^−1^, for E and 0.67 ± 0.58 to >10 mg mL^−1^ for G) and positive control (31.25 ± 0.00 to >125 ± 0.00 mg mL^−1^).

Analysis of the interaction among the monoterpenes obtained in the checkerboard assay (Table 3) showed synergism among all three monoterpenes, with the FICI values determined in the range 0.09–0.31, determined for the mixtures containing concentrations ≤ 1/4MIC of G, ≤1/16MIC of C and ≤1/4MIC of E.

### 2.2. Antibiofilm Potential

Concerning C, its higher applied concentrations (MIC/4–MIC) inhibited formation of biofilm by *E. faecium* (biofilm biomass reduction from 18.86% to 39.14%). Furthermore, C inhibited *K. pneumoniae* biofilm formation (42.08%, 33.14% and 34.52% reductions obtained at MIC/32, MIC/16 and MIC, respectively). In the case of *A. baumannii*, reduction in biofilm formation by C was observed at all tested concentrations except MIC, reaching up to 56.17%. Concerning *P. aeruginosa* and *Enterobacter* sp., inhibition of biofilm formation was observed only at MIC, valuing 66.35% and 39.65%, respectively. Finally, C showed no inhibitory effect against *S. aureus* MRSA; at higher tested concentrations (MIC/8–MIC), it even increased biofilm formation.

**Figure 1:**
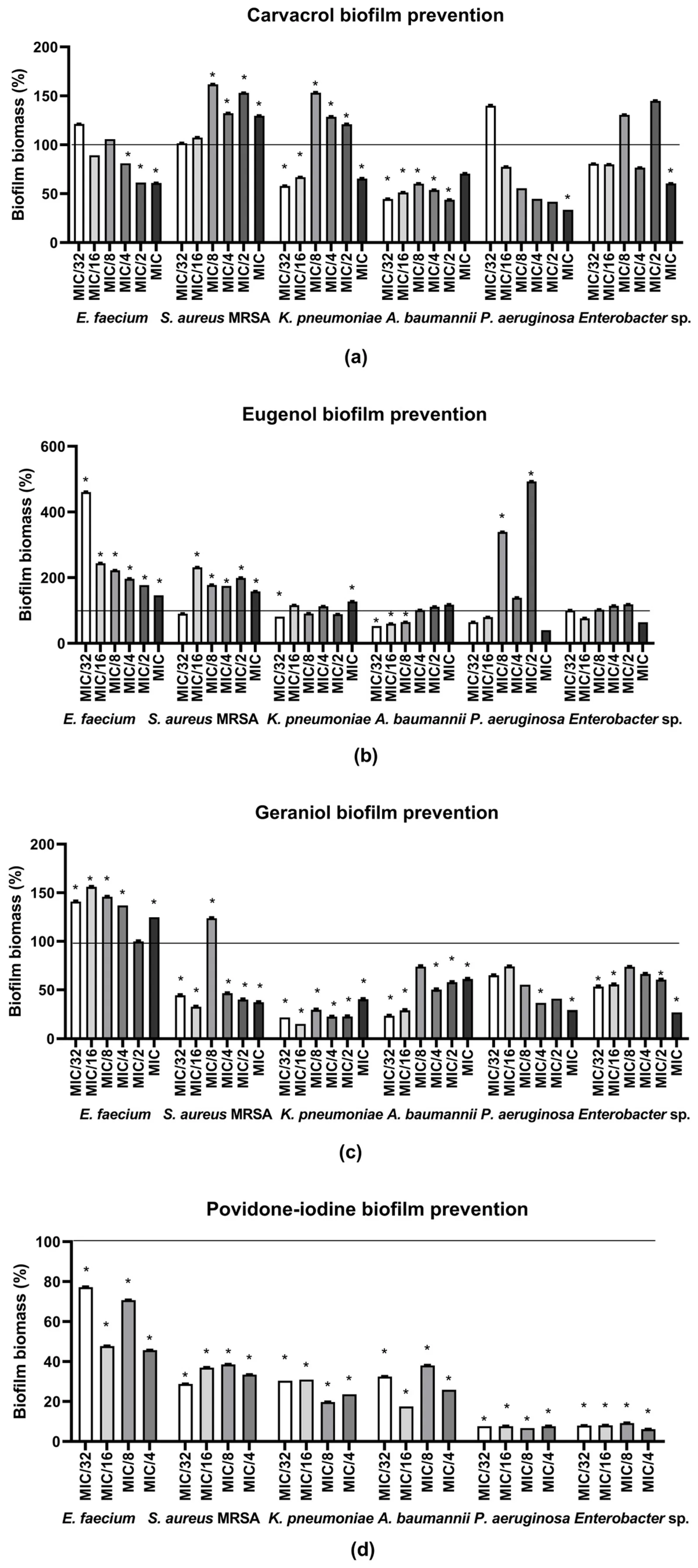
Potential of the selected agents to prevent the formation of biofilms of the ESKAPE pathogens: (**a**) carvacrol; (**b**) eugenol; (**c**) geraniol; (**d**) positive control (10% povidone-iodine solution). Presented values are percentages of formed biofilm biomass calculated with respect to the untreated control (100% biofilm biomass). G and C are abbreviations for geraniol and carvacrol, respectively. The results are expressed as the mean values ± standard deviation of two individual experiments, each performed in five replicates. Statistical significance was tested using one-way ANOVA: * Statistical difference in the biofilm biomass compared to the untreated control (100% biofilm biomass), *p* < 0.05.

Concerning E (Figure 1b), the only inhibitory potential was observed in the case of *A. baumannii* and *K. pneumoniae*, at MIC/32-MIC/8 and MIC/32 (35.38–47.14% and 18.85% inhibition), respectively. At all other concentrations and species, an increase in biofilm biomass production was observed.

G (Figure 1c) had a significant inhibitory effect on *K. pneumoniae* at all tested concentrations (MIC/32-MIC, within the range of 59.63–84.72% inhibition). Similar was observed in the case of *A. baumannii* and *Enterobacter* sp., with inhibitory ranges 38.78–76.39% and 39.49–72.93%, respectively. G had an inhibitory effect on *P. aeruginosa* biofilm formation at MIC/4 (63.37%) and MIC (70.71%). In the case of *S. aureus* MRSA, G induced prevention of biofilm formation at almost all tested concentrations (53.49–67.34% inhibition) except MIC/8, where it caused biofilm production. G at all tested concentrations showed upgrading effect on the *E. faecium* biofilm.

In order to valorize the monoterpenes’ potential to inhibit biofilm formation, a positive control, i.e., a solution of 10% povidone-iodine used for wound disinfection, was also screened. It has also significantly prevented the biofilm formation of all isolates in all tested concentrations: the inhibitions’ ranges were in the case of MIC/32 54.32% (*E. faecium*) 93.91% (*Enterobacter* sp.); MIC/16 29.27% (*E. faecium*) to 93.30% (*P. aeruginosa*); MIC/8 52.27% (*E. faecium*) to 92.36% (*P. aeruginosa*); MIC/4 22.81% (*E. faecium*) to 92.38% (*P. aeruginosa*) (Figure 1d).

**Figure 2:**
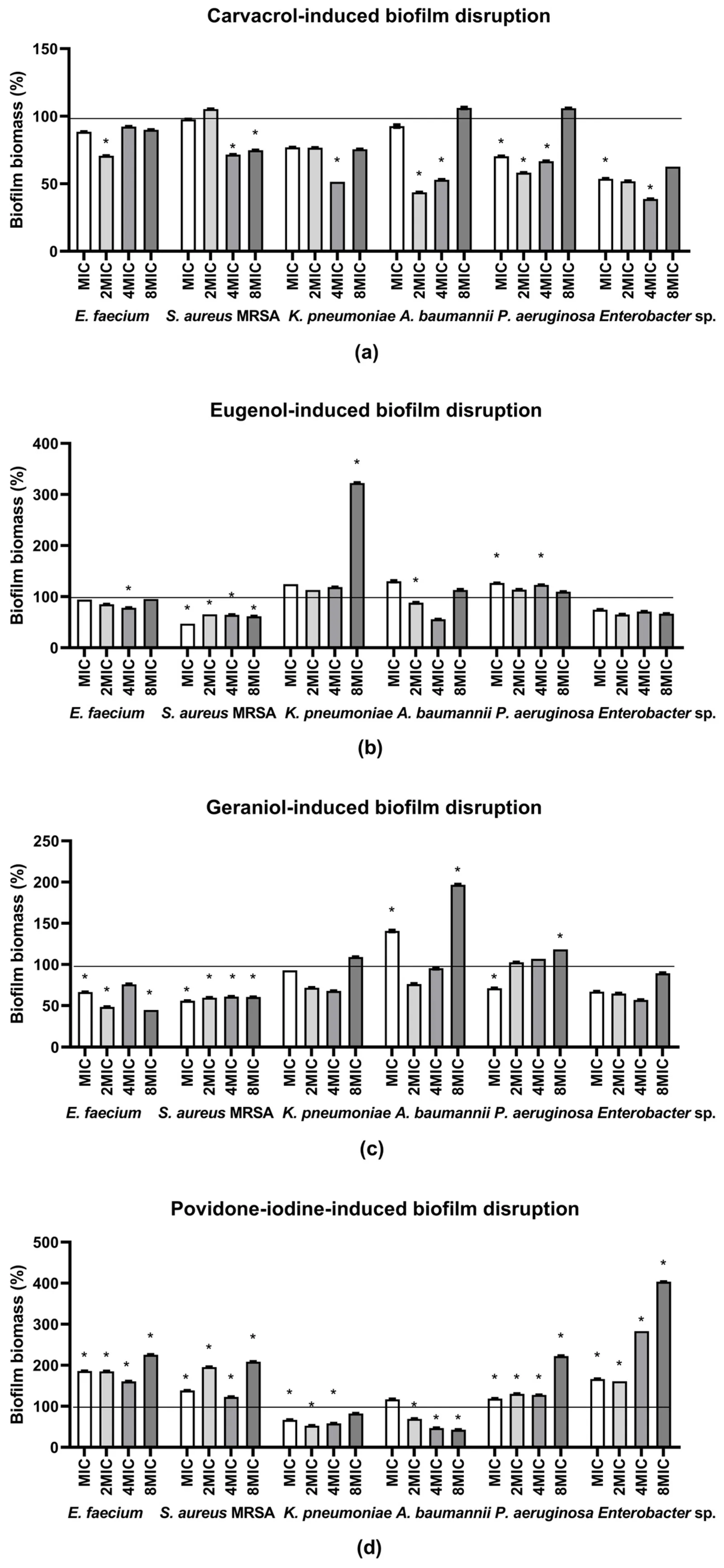
Potential of the selected agents to disrupt pre-formed biofilms of the ESKAPE pathogens: (**a**) carvacrol; (**b**) eugenol; (**c**) geraniol; (**d**) positive control (10% povidone-iodine solution). G and C are abbreviations for geraniol and carvacrol, respectively. The results are expressed as the mean values ± standard deviation of two individual experiments, each performed in five replicates. Statistical significance was tested using one-way ANOVA: * Statistical difference in the biofilm biomass compared to the untreated control (100% biofilm biomass), *p* < 0.05.

In the subsequent experiments, the disruptive potential of the monoterpenes against pre-formed biofilms was evaluated. Applied concentrations ranged from MIC to 8MIC. The activity of C tested on 48 h-old biofilms (Figure 2a) was heterogeneous. The highest susceptibility was observed in the case of *Enterobacter* sp., where significant biofilm reduction was detected at 4MIC (61.34%) and MIC (46.26%). A comparable disruption was recorded for *A. baumannii* at 2MIC (56.34%) and 4MIC (47.12%), as well as for *K. pneumoniae* at 4MIC (48.56%). Concerning *P. aeruginosa*, the disruption range was 29.63–41.77%, while the highest tested concentration increased overall biofilm biomass. In *S. aureus* MRSA, disruption was detected only at the highest concentrations tested (4MIC 28.42% and 8MIC 25.21%), but the overall effect remained low. Similarly, in *E. faecium*, biofilm eradication was observed exclusively at 2MIC.

E demonstrated the strongest antibiofilm activity against already formed biofilm of *S. aureus* MRSA across all tested concentrations, in the range of 34.41% (2MIC) to 52.83% (MIC) (Figure 2b). In contrast, eradicative effects against *E. faecium* (21.74%) and *A. baumannii* (44.32%) were detected only at 4MIC. No disruptive potential of E against pre-formed biofilms was observed in the remaining isolates.

G (Figure 2c) displayed pronounced antibiofilm activity against *E. faecium*, with the strongest inhibition recorded at 8MIC (55.26%) and 2MIC (51.49%), followed by MIC (33.52%). A slightly lower effect was observed in the case of *S. aureus* MRSA, where G had a consistently diminishing effect at all tested concentrations within the range of 38.98% to 44.09%, while the most effective was the lowest concentration. For *P. aeruginosa*, the lowest concentration tested resulted in the disruption of 28.83%, whereas the highest concentration induced biofilm upgrading. In *A. baumannii*, both the lowest and the highest tested concentrations stimulated further biofilm formation.

The activity of the positive control (10% povidone-iodine solution) against pre-formed biofilms was also evaluated (Figure 2d). Disruptive potential was observed only against *K. pneumoniae* and *A. baumannii*, with the most pronounced reduction detected at 2MIC (47.21%) and at 8MIC (57.12%), respectively. In all other cases, exposure to the positive control resulted in biofilm proliferation.

**Figure 3:**
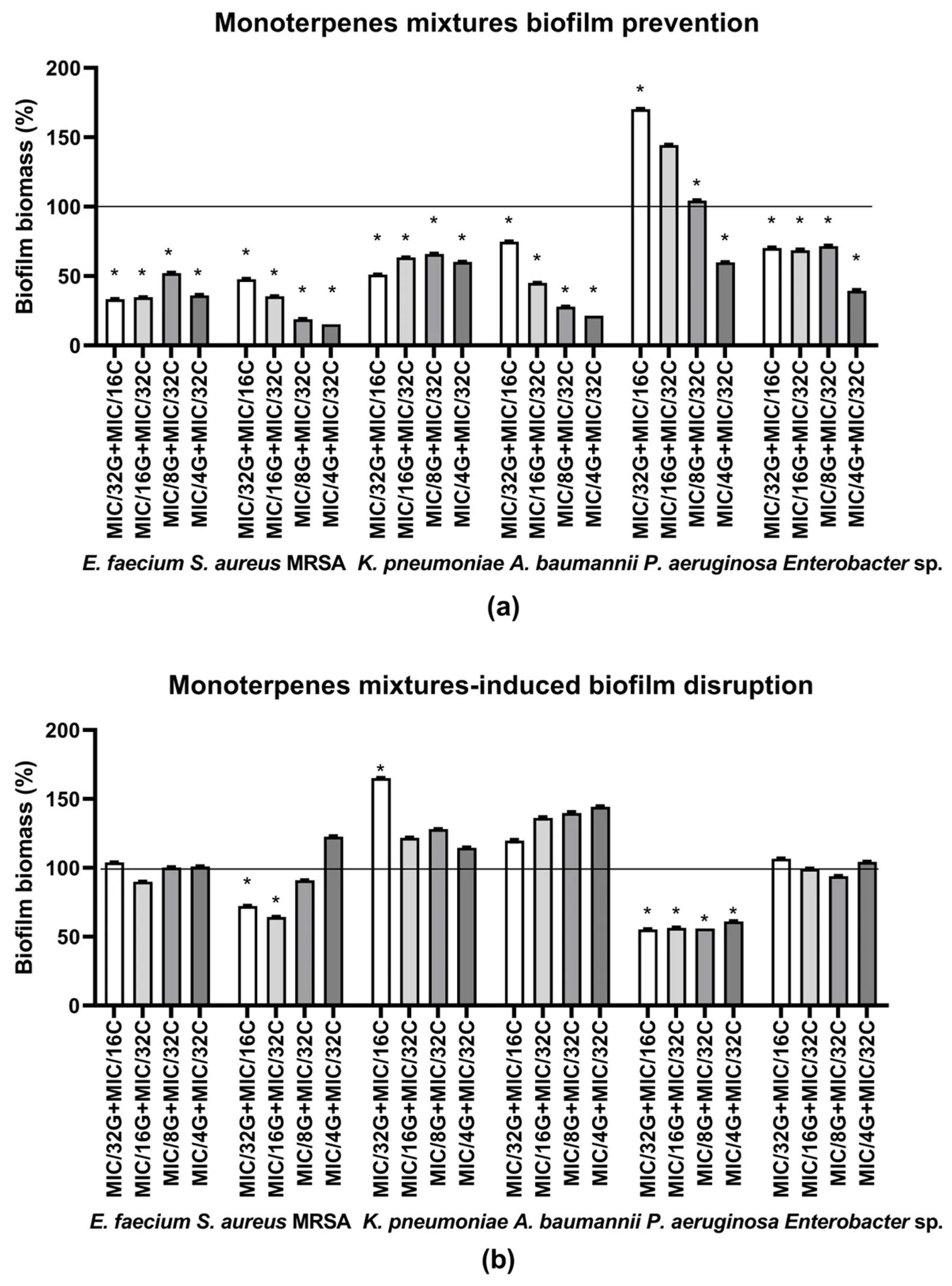
Potential of the monoterpenes’ mixture to prevent biofilm formation and to disrupt pre-formed biofilms of the ESKAPE pathogens: (**a**) monoterpenes mixture biofilm prevention; (**b**) monoterpenes mixture-induced disruption of biofilm. G and C are abbreviations for geraniol and carvacrol, respectively. The results are expressed as the mean values ± standard deviation of two individual experiments, each performed in five replicates. Statistical significance was tested using one-way ANOVA: * Statistical difference in the biofilm biomass compared to the untreated control (100% biofilm biomass), *p* < 0.05.

Considering the observed effects of individual monoterpenes, particularly the increased biofilm formation induced by E, it was of interest to investigate the combined effect of the other two monoterpenes. In accordance with the results given in Table 4, mixtures of the monoterpenes G and C providing synergistic antibacterial effects, i.e., (MIC/32 + MIC/16, MIC/16 + MIC/32, MIC/8 + MIC/32 and MIC/4 + MIC/32, respectively) were further tested, and their potential to prevent formation of biofilms and to eradicate already formed biofilms was shown in Figure 3. Results suggested that all four mixtures significantly prevented biofilm formation of all isolates, with the only exception of the *P. aeruginosa* biofilm, whose production was even stimulated when it was exposed to the mixtures with the lowest concentrations of G and C. The mixture MIC/32(G) + MIC/16(C) prevented biofilm formation of the isolates for 25.27% (*A. baumannii*) and 66.77% (*E. faecium*) (Figure 3a). A similar trend was observed with the mixture MIC/16(G) + MIC/32(C), where the inhibition range was from 31.40% (*Enterobacter* sp.) to 65.32% (*E. faecium*). In the case of the mixture containing MIC/8(G)+ MIC/32(C), inhibitory potential ranged from 28.34% (*Enterobacter sp*.) to 81.02% (*S. aureus* MRSA). The mixture with the highest concentration of G (MIC/4) was the most efficient, i.e., it inhibited biofilm formation in the range of 39.82–84.77% for *K. pneumoniae* and *S. aureus* MRSA, respectively.

The same G-C mixtures were also screened for the disruptive potential on pre-formed 48 h-old biofilms. Results obtained (Figure 3b) showed that all tested combinations significantly reduced biofilm biomass of *P. aeruginosa* only. The eradicative antibiofilm effect of all tested concentrations was relatively uniform (38.84–44.67%), while the most effective was the mixture MIC/32(G) + MIC/16(C). Biofilm biomass decrease was also observed in *S. aureus* MRSA at MIC/32(G) + MIC/16(C) (27.81%) and MIC/16(G) + MIC/32(C) (35.78%) concentrations. No disruptive effects were detected in the remaining strains.

Finally, in order to evaluate and visually compare the potential of sole monoterpenes and their mixtures to prevent biofilm formation, and consequently estimate whether the sole monoterpene’s activity was amplified, Table 4 summarizing the overall activity profiles was prepared. As shown, the combinations exhibited superior antibiofilm activity, compared to the single compounds, against *E. faecium*, *S. aureus* MRSA, *A. baumannii* and *Enterobacter* sp.

### 2.3. Cytotoxicity Assessment

**Figure 4:**
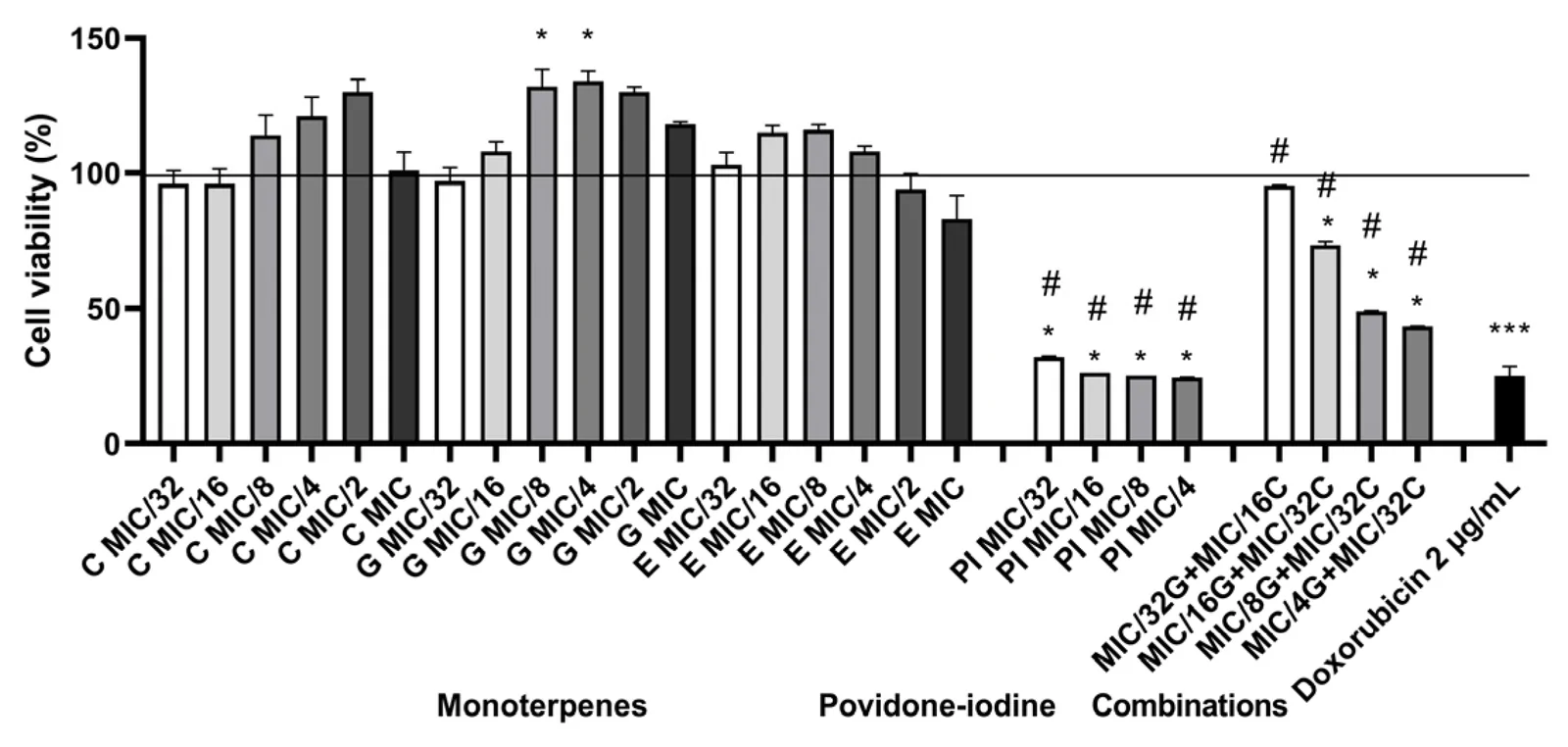
Cytotoxicity testing (XTT assay) of individual monoterpenes, different mixtures and 10% povidone-iodine dilutions. The results are expressed as the mean values ± standard deviations of two individual experiments, each performed in five replicates. Statistical significance was tested using the one-way ANOVA. * Statistical significance with respect to the untreated control (100% cell viability). # Statistical significance with respect to povidone-iodine (PI) treatment. */# *p* < 0.05; *** *p* < 0.001. MIC values of carvacrol, geraniol, eugenol and povidone-iodine were 0.31 mg mL^−1^, 3.63 mg mL^−1^, 2.69 mg mL^−1^ and 41.67 mg mL^−1^, respectively. # Statistical difference in cell viability compared to povidone-iodine (PI). G, C and PI are abbreviations for geraniol, carvacrol and povidone-iodine, respectively.

In order to provide a risk assessment of the use of the monoterpenes and their selected mixtures inducing promising antibiofilm potential, the cytotoxicity assay was applied. Tested monoterpene combinations were selected based on the results obtained from the checkerboard assay. The tested monoterpenes did not affect cell survival at any concentration that was active in the antibiofilm assay (Figure 4). Although the selected mixtures were cytotoxic, cell survival was significantly higher compared to a commonly used disinfectant (povidone-iodine). Moreover, the mixture with the lowest abundance of monoterpenes (MIC/32(G) + MIC/16(C)) was not cytotoxic.

## 3. Discussion

In this study, the antimicrobial and antibiofilm activity of G, E and C against MDR isolates belonging to all members of the ESKAPE group was demonstrated. Further, the interactions among the monoterpenes were explored, and a synergistic effect of binary combinations of all tested monoterpenes was observed. Furthermore, searching for the antibiofilm effect of sole monoterpenes pointed out the combination of G and C as the most promising for further research. Taking this into account, as well as the fact that G and C mixtures have not already been tested against biofilm, their mixtures, prepared at appropriate ratios inducing synergistic antibacterial activity, were investigated for their antibiofilm potential. In addition, the cytotoxicity of the G and C mixtures was screened in order to provide a preliminary assessment of their safety.

With respect to selected test organisms, it is worth noting that the literature data confirmed the ESKAPE group as the most commonly isolated from SSIs, suggesting also that 63% of recognized ESKAPE pathogens exhibited resistance to more than three antibiotic classes [15]. Among MDR isolates from SSIs, *E. faecium*, *S. aureus* MRSA, *K. pneumoniae*, *A. baumannii*, *P. aeruginosa* and *Enterobacter* sp. accounted for 5.2%, 43.5%, 29.6%, 5.2%, 13.4% and 3.5% of isolates, respectively [16]. Seid et al. [16] also demonstrated that the majority of ESKAPE pathogens isolated from SSIs were MDR, whereas a smaller proportion of *K. pneumoniae* isolates exhibited an XDR phenotype. The isolates tested in this study, i.e., *E. faecium* 5573-DM, *S. aureus* MRSA 3073-DM, *K. pneumoniae* 3625-DM, *A. baumannii* 3066-DM, *P. aeruginosa* 3082-DM, *Enterobacter* sp. 3071-DM were also derived from surgical site wounds. The majority of selected isolates in this study were XDR, namely *A. baumannii*, *P. aeruginosa* and *K. pneumoniae* being sensitive to colistin only and an *E. faecium* strain that was sensitive to linezolid only. On the other hand, the *S. aureus* MRSA isolate was denoted as MDR, while *Enterobacter* sp. 3071-DM was resistant to all tested antibiotics (PDR).

Compared to the positive control (10% povidone-iodine as a routine wound antiseptic), all tested monoterpenes showed significantly higher antimicrobial effects (*p* ˂ 0.05). Moreover, approximately 12, 16 and even 134 times higher antibacterial activity of G, E and C, respectively, was observed in comparison to that of povidone-iodine. To the best of our knowledge, for the first time, these monoterpenes were screened against multidrug-resistant strains in the following manner: G against *E. faecium*, *P. aeruginosa* and *K. pneumoniae*, and E and C against *Enterobacter* sp. Concerning the activity of G against *A. baumannii* (MIC 0.39 mg mL^−1^), the results obtained in this study were comparable with those presented by Choudhary et al. (MIC 0.49 mg mL^−1^) [17]. In addition, the sensitivity of *K. pneumoniae* to C (MIC 0.31 mg mL^−1^) and *E. faecium* (MIC 0.53 mg mL^−1^) was also comparable to the results of de Souza et al. (MIC 0.26 mg mL^−1^) [18] and Owen et al. (MIC 0.59 mg mL^−1^) [19], respectively. Marchese et al. [20] and Da Silva et al. [21] presented similar activities of C against *S. aureus* MRSA (MIC value 0.3 mg mL^−1^) and *P. aeruginosa* (MIC 0.128 mg mL^−1^), as in this study (MIC values 0.33 mg mL^−1^ and 0.16 mg mL^−1^, respectively). However, in other studies, the activity of G, E and C against multidrug-resistant isolates of the ESKAPE group differed in efficiency from the results presented in our study [22,23,24,25,26,27,28,29]. Latorre et al. [29] and Choudhary et al. [17] reported higher MICs of E against *A. baumannii* (1.92 and 1.00 mg mL^−1^, respectively) than those observed in the present study (0.34 mg mL^−1^). In contrast, Al-Shabib et al. [24] reported a lower MIC of E against *S. aureus* MRSA (0.66 mg mL^−1^) compared with our results (3.49 mg mL^−1^), while Selvaraj et al. [22] reported a higher MIC of C (0.50 mg mL^−1^) than that observed in the present study (0.33 mg mL^−1^). These discrepancies between the reported literature data and the findings of the present study may be attributed to differences in the origin of the isolates and, consequently, their genetic backgrounds. G is an acyclic monoterpene and its antibacterial mechanism involves an increase in membrane permeability, attributed to its interaction with lipids and other cellular membrane components [30,31]. In addition, Lin et al. [32] showed that G interacts with the cell wall of Gram-positive bacteria and induces its rupture. On the other hand, E and C are aromatic phenolic compounds, and their activity is attributed to hydrophobic properties and hydroxyl groups. The hydroxyl groups of E and C could interact with proteins and prevent enzymatic reactions, while high lipophilicity is responsible for the action on the cellular membrane [33]. However, although E is also a phenolic compound, its activity compared to C was rather lower, indicating that additional structural features determine the activity of each compound. In addition, C is more lipophilic than E, owing to its higher lipophilicity due to the absence of a methoxy group. This increased lipophilicity enhances its ability to bind to the cell membrane and increase its fluidity [33]. Furthermore, the hydroxyl group of C is directly attached to the phenolic ring, facilitating effective interactions with phospholipids and proteins. In contrast, it could probably be suggested that the presence of a methoxy group in E reduces its hydrogen-bonding potential, slightly diminishing its antimicrobial activity [33].

Taking into account the structural differences among monoterpenes, the idea of the study was that the monoterpenes may be used in combination in order to get higher antibacterial efficiency in comparison to their sole use. Such an idea is not new; actually, several already published studies used this strategy to enhance the overall antimicrobial activities of the essential oils and reach synergism [34,35]. To investigate this, an antibacterial checkerboard assay was utilized to identify the most effective monoterpene combinations with the lowest concentrations, thereby minimizing the risk of their potential cytotoxicity.

The third part of the study was dedicated to investigating the antibiofilm potential of the sole monoterpenes and their mixtures since SSI are biofilm-associated infections. Monoterpenes, involving G, C and E, exert their antibiofilm activity by reducing bacterial adhesion and synthesis of extracellular polymeric substance, disrupting hydrophobic interactions inside the exopolysaccharide matrix, and suppressing quorum sensing pathways [36,37,38]. Concerning the range of antibiofilm activity observed in this study, it is worth noting that there is available data concerning the individual effect of G, C and E on ESKAPE pathogens’ biofilm inhibition, but not always on the biofilms of multidrug-resistant isolates, especially those of surgical wound origin. Regarding the inhibitory potential against biofilm formation, available literature data pointed out that G is effective against *S. aureus* MRSA [39] and MDR *A. baumannii* [40]. In addition, biofilm formation was prevented with C in the case of MDR *A. baumannii* and *K. pneumoniae* [41,42] and with E treatment of *A. baumannii* [43], *K. pneumoniae* [44], MRSA [28] and *P. aeruginosa* [45]. As far as we know, antibiofilm potential, i.e., inhibition of formation, against (1) G against *K. pneumoniae*, *P. aeruginosa*, *E. faecium* and *Enterobacter* sp., (2) C against *S. aureus* MRSA, *E. faecium*, *P*. *aeruginosa* and *Enterobacter* sp. and (3) E against *E. faecium* and *Enterobacter* sp., all referring to the multidrug-resistant strains, was evaluated for the first time in this study.

Regarding the disruption of pre-formed biofilms, available data suggest that, to the best of our knowledge, this study is the first to evaluate the effect on multidrug-resistant strains, namely of (1) G against *E. faecium*, *K. pneumoniae*, *P. aeruginosa* and *Enterobacter* sp., (2) C against *E. faecium* and *Enterobacter* sp. and (3) E against *E. faecium*, *P. aeruginosa* and *Enterobacter* sp. Our results are in line with Sreepian et al. [46] who reported the efficacy of G in disrupting *S. aureus* MRSA biofilms, but are opposite to Aslan & Alim [47] reporting an effect on MDR *A. baumannii* (no inhibition was detected in our study). Kashi et al. [48,49] also showed an effect of C on MDR *A. baumannii* and *K. pneumoniae*, while Nikolic et al. [50] reported an even higher disruptive effect of C on pre-formed *S. aureus* MRSA biofilms. Regarding E, Yadav et al. [28] and Kashi et al. [48] showed eradication of *S. aureus* MRSA and MDR *A. baumannii* biofilms, respectively, being consistent with this study. Although E did not reduce pre-formed biofilms of *K. pneumoniae* in this study, Kashi et al. [49] reported the opposite result. The discrepancies between the present findings and those reported in the literature may be attributed to differences in the origin and genetic background of the bacterial isolates. Furthermore, the use of reference rather than clinical MDR strains in some previous studies may have contributed to the observed differences in biofilm susceptibility.

Concerning the observed pattern of all monoterpenes’ activity, it is interesting to note that the curves of both antibiofilm effects, i.e., formation inhibition and eradication of already formed biofilm, were rather (inverted) U-shaped rather than linear (dose-dependent). Actually, in the cases of several pathogens and treatments, such as in the inhibition of biofilm formation of *E. faecium*, *K. pneumoniae* and *P. aeruginosa* with G, *P. aeruginosa* and *Enterobacter* sp. with E and *A. baumannii* and *Enterobacter* sp. with C, the medium applied concentrations induced the most pronounced effect. Such a hormetic response could be explained as follows: lower tested concentrations actually act as mild stressors, triggering an adaptive response, i.e., induction of biofilm formation as an attempt by bacteria to protect themselves from antibacterial substances [51,52]. While medium concentrations induce the highest antibiofilm activity, the highest ones result in a decrease in activity mainly due to possible aggregation of test substances, which consequently reduces bioavailability and possibly blocks penetration through the biofilm matrix [53]. A similar response has already been established for different antimicrobial substances. Hoffman et al. [54] have shown that aminoglycosides applied in subinhibitory concentrations actually induce an adaptive response and enhance biofilm formation in *P. aeruginosa* and *E coli*, while Jones et al. [55] pointed out that an adaptive response, i.e., increased expression of the type VI secretion system in *P. aeruginosa*, has been induced by subinhibitory concentrations of kanamycin. In addition, subinhibitory concentrations of antimicrobial peptides could also induce an adaptive response [56]. Finally, natural products of plant origin are not exceptions; actually, the antibiofilm effect of *Eugenia brejoensis* essential oil against *A. baumannii* [57] and *Frangula emodin* against *S. aureus* [58] exhibited a nonlinear dose–response effect, resembling an inverted U-shaped response.

Given that E was found to induce biofilm formation in the majority of tested strains, only G-C combinations demonstrating synergistic activity in the antibacterial checkerboard assay were selected for evaluation of their antibiofilm activity. To our knowledge, a mixture of G and C was tested for the first time in our study on ESKAPE pathogens. Concerning the mixtures’ antibiofilm potential, it is worth noting that the most common manner to analyze the type of interaction between test substances is by calculation of the minimum biofilm inhibition/eradication concentration 50% used for FICI determination [59,60]. However, a dose-dependent response of test strains to the sole test substances is necessary to quantify FICI values and clearly determine the type of interaction between the substances. Taking into account that the obtained response in the antibiofilm assay performed with sole monoterpenes was dominantly U-shaped, this methodology could not be applied. However, thorough comparative analysis of the biofilm biomass reduction values obtained in the experiments determining biofilm formation inhibition (Table 4) could provide some information concerning types of interaction. Indeed, in the case of *E. faecium*, only the G and C mixture induced notable antibiofilm activity, while the sole monoterpenes stimulated biofilm formation (G) or had no effect (C), indicating some positive interaction between the substances. Similar was obtained in the case of combinations containing MIC/8 and MIC/4 of G influencing *S. aureus* MRSA, *A. baumannii* and *Enterobacter* sp. biofilms. However, in the case of all combination treatments of *K. pneumoniae* and *P. aeruginosa*, as well as in the case of combinations with lower G concentrations (MIC/32 and MIC/16) affecting biofilms of *A. baumannii* and *Enterobacter* sp., antagonistic activity between monoterpenes was observed. Such a response being species- and concentration-dependent indicates a complex type of interaction between G and C and should be further investigated. On the other hand, it is worth noting that the same mixtures of monoterpenes’ inhibitory concentrations have even eradicated already formed biofilm (Figure 3b), clearly indicating the enhancement of antibiofilm activity by the monoterpenes mixing.

The main form of prevention of SSIs is the use of antiseptics, and the most common antiseptic is 10% povidone-iodine. This widely used antiseptic exhibits rapid antimicrobial activity by targeting multiple cellular sites, distinguishing it from antibiotics [9]. However, iodine may induce acute pain, irritation and staining at the site of use [9]. Additionally, iodine has the potential to form complexes with organic matter, thereby reducing its bactericidal efficacy [61]. In our study, povidone-iodine was used as a commonly used disinfectant in the antibacterial and antibiofilm assays, and even though it was effective, the effective concentrations were much higher compared to tested mixtures of G and C.

Following the evaluation of the antibiofilm activity of the monoterpenes and their mixtures, the final objective of this study was to assess their safety. To achieve this, a cytotoxicity assay was conducted, solely for monoterpenes, but also for the mixtures. It was previously suggested that all three monoterpenes possess some cytotoxic potential [62]. However, our results suggested that monoterpenes at concentrations tested in this study did not affect cell viability. Similar was suggested by Rodenak-Kladniew et al. [63] who investigated the cytotoxic effects of G at concentrations ranging from 0.04 to 0.19 mg mL^−1^ and concluded that G did not significantly inhibit cell viability, exhibiting only low cytotoxicity. The possible explanation could be seen in the fact that cytotoxicity of the aforementioned monoterpenes depends on the applied concentrations as well as the specific cell line used in the assay. In addition, in order to screen for the effect of possible interactions which could affect human cells’ viability, cytotoxicity assay was also applied with the most efficient antibiofilm combinations of the G and C. Obtained results demonstrated that the used mixtures, especially the one containing the lowest tested concentrations of monoterpenes (MIC/32(G) + MIC/16 (C)) carry a lower risk of injury to human cells compared to the conventional povidone-iodine. Based on these findings, this mixture could be considered a valuable one to enhance efficacy at lower concentrations of the monoterpenes, attributed to the synergistic interaction between G and C.

To the best of our knowledge, the monoterpenes investigated in the present study are not approved as therapeutic agents for the treatment of wounds. Therefore, our pioneering findings should be considered as preliminary in vitro evidence of their potential antibacterial and antibiofilm activity against MDR ESKAPE pathogens. Further in vivo and clinical studies, as well as appropriate safety and regulatory evaluations, are required before these compounds or their combinations can be considered for therapeutic application in clinical practice.

## 4. Materials and Methods

### 4.1. Monoterpenes

Pure monoterpenes (˃97%): carvacrol (CAS No. 499-75-2, Sigma-Aldrich, St. Louis, MI, USA), eugenol (CAS No. 97-53-0, Thermo Scientific, Waltham, MA, USA) and geraniol (CAS No. 106-24-1, Sigma-Aldrich, St. Louis, MI, USA) were used.

### 4.2. Bacterial Strains, Identification and Antimicrobial Susceptibility Screening and Ethical Approval

This study was conducted in accordance with the Declaration of Helsinki, with approval from the Local Ethical Committee of Clinical Hospital Center “Dr. Dragiša Mišović-Dedinje” (No 7215/8-2026). Clinical isolates tested in the study originated from patients of the Surgical Department of University Hospital Center “Dr. Dragiša Mišović-Dedinje”, however, the study was not a clinical trial, and bacteria were collected as part of the standard procedure in the hospital (they were identified as a common procedure in the treatment of the patients within the Hospital). No personal data or other data from the patients were collected, and informed consent was not required.

Identification and antimicrobial susceptibility testing were performed using the Vitek 2 system (bioMérieux, Marcy-l’Étoile, France), as it is routinely used within the “Dr. Dragiša Mišović-Dedinje” University Hospital Center as part of diagnostic procedures. Upon receipt in the laboratory, clinical samples were inoculated onto Columbia blood agar supplemented with 5% sheep blood and CPSE agar (bioMérieux, Marcy-l’Étoile, France) and incubated for 18–24 h. Following incubation, pure colonies were selected for identification and antimicrobial susceptibility testing using the Vitek 2 system. The collection of isolates was carried out from March 2025 to August 2025 and clinical isolates used in this study were *E. faecium* (5573-DM), *S. aureus* MRSA (3073-DM), *K. pneumoniae* (3625-DM), *A. baumannii* (3066-DM), *P. aeruginosa* (3082-DM) and *Enterobacter* sp. (3071-DM).

Concerning antimicrobial susceptibility, antibiotic selection for each identified bacterium is in line with the intrinsic resistance profiles. Since it is not identical among bacterial species, the number and spectrum of antimicrobial agents tested differed accordingly. Breakpoints from the European Committee on Antimicrobial Susceptibility Testing (EUCAST, version 15.0, 2025) were used for determining bacterial resistance.

After identification and characterization, strains were stored at −80 °C in the Microbiology Department of the “Vinča’’ Institute—Institute for Nuclear Sciences, University of Belgrade.

### 4.3. Bacterial Cultivation

The bacterial cultivation was carried out by streaking frozen stock cultures onto Mueller-Hinton agar plates (MHA, Torlak, Belgrade, Serbia) followed by 48 h incubation at 37 °C.

The bacterial inoculum used for all further tests was adjusted to be 0.5 McFarland, corresponding to 1 × 10^8^ CFU mL^−1^. All microbial procedures were carried out in a sterile environment.

### 4.4. The Assessment of the Antibacterial Potential

The antibacterial potential of the monoterpenes was examined by the use of the broth-microdilution assay. Minimal inhibitory concentrations (MICs) as well as minimal bactericidal concentrations (MBCs) were determined according to the method previously outlined by Vasilijević et al. [64] with slight modifications. Briefly, monoterpenes were prepared by dissolving them in Tween 80 (G: Tween 80 1:1, E: Tween 80 1:1, C: Tween 80 1:2). Concentration ranges were 0.21–13.50 mg mL^−1^, 0.08–10.80 mg mL^−1^ and 0.06–8.40 mg mL^−1^ for E, G and C, respectively. The negative control was the solvent Tween 80 (1.66–106 mg mL^−1^), while a positive control was a commercial solution of 10% povidone-iodine (tested concentration range 0.98–125 mg mL^−1^). Bacterial inoculum was added to each well to achieve a final concentration of 10^5^ CFU mL^−1^. MIC values were determined in accordance with the color changes of the growth indicator resazurin. MBCs were determined by plating from the wells with no noticeable growth onto Mueller-Hinton agar plates at 37 °C for 24 h under aerobic conditions. The experiment was performed in triplicate and repeated two times.

### 4.5. Antibacterial Checkerboard Assay

The checkerboard assay was performed as previously described in Vasilijević et al. [64]. Briefly, the tested concentration range of the substances was (1/32) × MIC − 2 × MIC. All combinations that did not induce a color change of resazurin (lack of growth) were used for the calculation of the fractional inhibitory concentration index (FICI) for two antimicrobials in combination. The FICI was calculated according to Equation (1),

(1) $$FICI=\frac{MICA\;in\;comb.}{MICA\;alone}+\frac{MICB\;in\;comb.}{MICB\;alone}$$

where substances A and B were monoterpenes.

FICI was used to distinguish between the modes of interaction as follows: FICI ≤ 0.5—synergistic; 0.5 < FICI ≤ 1—additive; 1 < FICI ≤ 4—indifferent; FICI > 4—antagonistic effect. The checkerboard assay was performed in triplicate in two individual experiments.

### 4.6. The Assessment of the Antibiofilm Activity of the Monoterpenes and Selected Mixtures

The impact of antibacterial agents on biofilm formation was determined in 96-well microtiter plates according to the previously described method in Marinkovic et al. [65]. The biofilms were formed during 24 h at 37 °C from the bacterial inocula of 10^5^ CFU/well, with or without sole monoterpenes (tested concentration ranges MIC/32—MIC), or G-C mixtures. Selection of these two monoterpenes, as well as of their concentrations tested for the combination activity, was carried out in respect of the antibiofilm potential of individual monoterpenes and the results of the antibacterial checkerboard assay.

On the other hand, in the biofilm disruption assay, the bacterial cell suspensions (2 × 10^5^ CFU/well) were first incubated for 48 h in order to allow biofilm formation. Pre-formed biofilms were exposed for 24 h to the tested monoterpenes (tested concentrations range MIC-8MIC). In addition, the eradication potential of the monoterpene mixtures (the same concentrations were combined as for the biofilm formation inhibition experiments) was also determined.

Biofilm biomass quantification was provided for both the inhibition of biofilm formation and pre-formed biofilm eradication using crystal violet (0.1%). The percentage of the biofilm biomass reduction (R), obtained as a result of inhibition of biofilm formation or pre-formed biofilm eradication, was calculated according to Equation (2):

(2) $$R=\left(1-\frac{At}{Ac}\right)\times 100\%$$

Ac and At are absorbances at 570 nm of control and treatment, respectively. The experiment was performed in five replicates and repeated two times.

### 4.7. Cytotoxicity Assessment

#### Cell Culture and Treatment Within XTT Assay

Cytotoxicity testing of G and C mixtures was performed on the normal human lung fibroblast cell line MRC-5 (ATCC, Manassas, VA, USA ECACC 84101801), according to the standard procedures [66]. The MRC-5 cell line was selected for cytotoxicity assessment in this study, as it is widely used as a model system for in vitro toxicity studies [67] due to its ability to undergo continuous growth and proliferation while maintaining a stable normal diploid karyotype and fibroblast-like morphology [68]. Ratios of the monoterpenes in the tested mixtures were determined in accordance with the checkerboard assay, as the most effective synergistic mixtures against the tested bacteria.

The cell cultures were treated with each monoterpene and the selected mixtures for 24 h. Untreated cells were used as a negative control, while 10% povidone-iodine was used to compare the cytotoxicity of the tested monoterpene mixtures with the same effect of already approved conventional disinfectant. For the positive control, doxorubicin (2 µg mL^−1^) was used. All treatments were set up in triplicate and repeated twice. The results are presented as a percentage of viable cells compared to the negative (untreated) control (100% of viable cells).

### 4.8. Statistical Analysis

Results are presented as mean value ± standard error of the mean (SEM). Statistical analysis was performed using the one-way ANOVA test in SPSS 10 (IBM, Armonk, NY, USA), followed by Tukey’s post hoc test for multiple comparisons. *p* values < 0.05 were accepted as the level of significance.

## 5. Conclusions

This work includes an investigation of the antibacterial and antibiofilm effects of selected monoterpenes G, C and E on clinical isolates of the ESKAPE group of pathogens. All tested strains originated from infected surgical sites. Isolates of *S. aureus* MRSA were MDR; *E. faecium*, *A. baumannii*, *P. aeruginosa* and *K. pneumoniae* were XDR, and *Enterobacter* spp. were PDR. All monoterpenes induced notable antibacterial effects, with C being the most potent. Some binary combinations of all tested monoterpenes showed synergistic antibacterial activity. Prevention of biofilm formation by all ESKAPE isolates was observed for G and C, while their mixtures exhibited antibiofilm activity against *E. faecium*, *S. aureus* MRSA, *A*. *baumannii* and *Enterobacter* sp. Cytotoxicity screening indicated a lower cytotoxic risk associated with the use of monoterpenes and their mixtures compared with conventional povidone-iodine treatment.

For the first time, this study comprehensively evaluates the antibacterial and antibiofilm potential of the selected monoterpenes against MDR ESKAPE pathogens, with particular emphasis on the activity of their binary combinations. Accordingly, considering all the above-mentioned findings, the obtained results may be considered an important step forward with potentially significant clinical implications. For that reason, further investigation is strongly encouraged, especially in order to determine the mechanism underlying enhancement of antibacterial and antibiofilm activities of the monoterpenes’ mixtures.

## Figures and Tables

**Table 1:** Sensitivity pattern of isolates belonging to the ESKAPE group.

**Isolate 5573-DM: *Enterococcus faecium* VRE**			
**Antibiotics**	**MIC**	**Interpretation**	**Group of Antibiotics**
Ampicillin	>8	R	Penicillins
Imipenem	>8	R	Carbapenems
Gentamicin SYN	>500	R	Aminoglycosides
Ciprofloxacin	>4	R	Fluoroquinolones
Levofloxacin	>4	R	Fluoroquinolones
Vancomycin	>4	R	Glycopeptides
Linezolid	2.00	S	Oxazolidinones
Antibiotic resistance level: XDR			
**Isolate 3073-DM:** ***Staphylococcus aureus*** **MRSA**			
Benzylpenicillin	>0.25	R	Penicillins
Ampicillin	>8	R	Penicillins
Oxacillin *	>2	R	Penicillins
Amikacin	≤8	S	Aminoglycosides
Gentamicin	>8	R	Aminoglycosides
Ciprofloxacin	≤0.5	I	Fluoroquinolones
Levofloxacin	≤0.5	I	Fluoroquinolones
Moxifloxacin	≤0.25	S	Fluoroquinolones
Erythromycin	>2	R	Macrolides
Clindamycin	>1	R	Lincosamides
Tetracycline	≤0.25	S	Tetracyclines
Vancomycin	1.00	S	Glycopeptides
Linezolid	2.00	S	Oxazolidinones
Trimethoprim-sulfamethoxazole	20.0	S	Sulfonamides
Antibiotic resistance level: MDR			
**Isolate 3625-DM:** ***Klebsiella pneumoniae***			
Ampicillin	>16	R	Penicillins
Amoxicillin-clavulanic acid	>32	R	Penicillins
Piperacillin-tazobactam	>64	R	Penicillins
Cefotaxime	>32	R	Cephalosporins
Ceftriaxone	>32	R	Cephalosporins
Ceftazidime	>32	R	Cephalosporins
Cefepime	>16	R	Cephalosporins
Imipenem	>8	R	Carbapenems
Meropenem	>8	R	Carbapenems
Ertapenem	>8	R	Carbapenems
Amikacin	>32	R	Aminoglycosides
Gentamicin	>8	R	Aminoglycosides
Tobramycin	>8	R	Aminoglycosides
Ciprofloxacin	>2	R	Fluoroquinolones
Levofloxacin	>4	R	Fluoroquinolones
Colistin	0.5	S	Polymyxin B
Antibiotic resistance level: XDR			
**Isolate 3066-DM:** ***Acinetobacter baumannii***			
Imipenem	>8	R	Carbapenems
Meropenem	>8	R	Carbapenems
Amikacin	>16	R	Aminoglycosides
Gentamicin	>4	R	Aminoglycosides
Tobramycin	>4	R	Aminoglycosides
Ciprofloxacin	>1	R	Fluoroquinolones
Levofloxacin	>2	R	Fluoroquinolones
Colistin	≤1	S	Polymyxin B
Antibiotic resistance level: XDR			
**Isolate 3082-DM:** ***Pseudomonas aeruginosa***			
Piperacillin-tazobactam	>64	R	Penicillins
Ceftazidime	>32	R	Cephalosporins
Cefepime	>16	R	Cephalosporins
Imipenem	>8	R	Carbapenems
Meropenem	>8	R	Carbapenems
Amikacin	>32	R	Aminoglycosides
Tobramycin	>8	R	Aminoglycosides
Ciprofloxacin	>2	R	Fluoroquinolones
Levofloxacin	>4	R	Fluoroquinolones
Colistin	0.5	S	Polymyxin B
Antibiotic resistance level: XDR			
**Isolate 3071-DM:** ***Enterobacter*** **sp.**			
Ampicillin	>16	R	Penicillins
Amoxicillin-clavulanic acid	>32	R	Penicillins
Piperacillin-tazobactam	>64	R	Penicillins
Cefalexin	>32	R	Cephalosporins
Ceftriaxone	>32	R	Cephalosporins
Ceftazidime	>32	R	Cephalosporins
Cefepime	>16	R	Cephalosporins
Imipenem	>8	R	Carbapenems
Meropenem	>8	R	Carbapenems
Ertapenem	>8	R	Carbapenems
Amikacin	>32	R	Aminoglycosides
Gentamicin	>8	R	Aminoglycosides
Tobramycin	>8	R	Aminoglycosides
Ciprofloxacin	>2	R	Fluoroquinolones
Levofloxacin	>4	R	Fluoroquinolones
Trimethoprim-sulfamethoxazole	>160	R	Sulfonamides
Antibiotic resistance level: PDR			

* Oxacillin replaces methicillin in clinical use. The antibiotic panel was selected according to the recommendations of the antibiotics being routinely tested for each bacterial pathogen (EUCAST, version 15.0, 2025).

**Table 2:** Antibacterial potential of monoterpenes against isolates belonging to the ESKAPE group.

	Carvacrol		Eugenol		Geraniol		Povidone-Iodine	
	MIC	MBC	MIC	MBC	MIC	MBC	MIC	MBC
	mg mL^−1^							
*E. faecium*	**0.53 ± 0.00 ^A^**	0.53 ± 0.00	**3.49 ± 1.86**	13.50 ± 0.00	**4.05 ± 1.66**	5.63 ± 1.94	31.25 ± 0.00	31.25 ± 0.00
*S. aureus MRSA*	0.33 ± 0.13	1.06 ± 0.00	**3.49 ± 1.86**	5.63 ± 1.94	2.36 ± 0.58	5.17 ± 1.70	31.25 ± 0.00	31.25 ± 0.00
*K. pneumoniae*	0.31 ± 0.12	0.53 ± 0.00	**5.17 ± 1.70**	8.55 ± 4.34	**3.49 ± 1.86**	4.38 ± 1.44	62.50 ± 0.00	62.50 ± 0.00
*A. baumannii*	0.35 ± 0.15	0.53 ± 0.00	0.34 ± 0.00 ^B^	0.68 ± 0.00	0.39 ± 0.00	0.78 ± 0.00	31.25 ± 0.00	31.25 ± 0.00
*P. aeruginosa*	0.16 ± 0.07	0.26 ± 0.00	1.13 ± 0.39	1.35 ± 0.00	0.67 ±0.58	0.67 ±0.58	31.25 ± 0.00	31.25 ± 0.00
*Enterobacter* sp.	0.20 ± 0.11	0.26 ± 0.00	2.50 ± 0.00	5.00 ± 0.00	**10.8 ± 0**	˃10	62.50 ± 0.00	˃125 ± 0.00
	Average MIC		Average MIC		Average MIC		Average MIC	
	0.31 ± 0.24 ^C^		2.69 ± 2.10		3.63 ± 3.08		41.67 ± 16.70	

^A^ Bolded numbers correspond to the highest MIC values; ^B^ Underlined numbers correspond to the lowest MIC values. ^C^ The assessment of antibacterial activity was performed in two independent experiments, with three technical replicates in each experiment.

**Table 3:** Type of interaction among the constituents.

**Geraniol (MIC)**	**Carvacrol (MIC)**	**FICI**	**Interpretation**
2	1/32	2.03125	Indifferent
1	1/32	1.03125	Indifferent
½	1/32	0.53125	Additive
¼	1/32	0.28125	Synergistic
1/8	1/32	0.15625	Synergistic
1/16	1/32	0.09375	Synergistic
1/32	1/16	0.09375	Synergistic
Eugenol (MIC)	Carvacrol (MIC)	FICI	Interpretation
2	1/16	2.0625	Indifferent
1	1/16	1.0625	Indifferent
½	1/16	0.5625	Additive
¼	1/16	0.3125	Synergistic
1/8	1/16	0.1875	Synergistic
1/16	1/16	0.125	Synergistic
1/32	1/16	0.09375	Synergistic
Eugenol (MIC)	Geraniol (MIC)	FICI	Interpretation
2	1/16	2.0625	Indifferent
1	1/16	1.0625	Indifferent
½	1/16	0.5625	Additive
¼	1/16	0.3125	Synergistic
1/8	1/16	0.1875	Synergistic
1/16	1/16	0.125	Synergistic
1/32	1/16	0.09375	Synergistic

The type of interaction expressed by FICI values is considered to be synergistic if FICI ≤ 0.5, additive if 0.5 < FICI ≤ 1, indifferent if 1 < FICI ≤ 4 and antagonistic if FICI > 4.0.

**Table 4:** Effect of sole monoterpenes and their combinations on biofilm formation.

Biofilm Formation					
G		C		G−C	
*E. faecium*					
**Conc.**	**% R ^A^**	**Conc.**	**% R**	**Conc.**	**% R**
MIC/32	−40.97	MIC/16	n.d. ^B^	MIC/32 + MIC/16	66.77
MIC/16	−56.03	MIC/32	n.d.	MIC/16 + MIC/32	65.32
MIC/8	−45.85	MIC/32	n.d.	MIC/8 + MIC/32	47.98
MIC/4	−36.94	MIC/32	n.d.	MIC/4 + MIC/32	63.93
*S. aureus* MRSA					
MIC/32	55.54	MIC/16	n.d.	MIC/32 + MIC/16	52.27
MIC/16	67.34	MIC/32	n.d.	MIC/16 + MIC/32	64.59
MIC/8	−23.69	MIC/32	n.d.	MIC/8 + MIC/32	81.02
MIC/4	53.49	MIC/32	n.d.	MIC/4 + MIC/32	84.77
*K. pneumoniae*					
MIC/32	78.06	MIC/16	33.14	MIC/32 + MIC/16	48.98
MIC/16	84.72	MIC/32	42.08	MIC/16 + MIC/32	36.60
MIC/8	70.38	MIC/32	42.08	MIC/8 + MIC/32	34.02
MIC/4	77.26	MIC/32	42.08	MIC/4 + MIC/32	39.82
*A. baumannii*					
MIC/32	76.39	MIC/16	48.68	MIC/32 + MIC/16	25.27
MIC/16	70.85	MIC/32	55.39	MIC/16 + MIC/32	55.00
MIC/8	n.d.	MIC/32	55.39	MIC/8 + MIC/32	72.16
MIC/4	49.72	MIC/32	55.39	MIC/4 + MIC/32	78.53
*P. aeruginosa*					
MIC/32	n.d.	MIC/16	n.d.	MIC/32 + MIC/16	−70.19
MIC/16	n.d.	MIC/32	n.d.	MIC/16 + MIC/32	−44.46
MIC/8	n.d.	MIC/32	n.d.	MIC/8 + MIC/32	−4.45
MIC/4	63.37	MIC/32	n.d.	MIC/4 + MIC/32	40.17
*Enterobacter* sp.					
MIC/32	46.56	MIC/16	n.d.	MIC/32 + MIC/16	29.77
MIC/16	44.48	MIC/32	n.d.	MIC/16 + MIC/32	31.40
MIC/8	n.d.	MIC/32	n.d.	MIC/8 + MIC/32	28.34
MIC/4	n.d.	MIC/32	n.d.	MIC/4 + MIC/32	60.57

^A^ %R–reduction of biofilm biomass. Negative percentages refer to the cases in which the biofilm biomass exceeded 100%, indicating that the monoterpenes or their combinations stimulated biofilm formation. ^B^ n.d.–no statistically significant difference detected.

## Data Availability

The raw data supporting the conclusions of this article are available.

## Abbreviations

C	Carvacrol
CFU	Colony-forming units
Conc.	Concentration
E	Eugenol
EOs	Essential oils
FICI	Fractional inhibitory concentration index
G	Geraniol
G-C	Geraniol-carvacrol mixture
MBC	Minimal bactericidal concentration
MDR	Multidrug-resistant pathogens
MHA	Mueller–Hinton Agar
MIC	Minimal inhibitory concentration
MRSA	Methicillin-resistant *Staphylococcus aureus*
n.d.	Not statistically significant difference detected
PDR	Pandrug-resistant bacteria
PI	Povidone-iodine
R%	Reduction of biofilm biomass
SSIs	Surgical site infections
VRE	Vancomycin-resistant *Enterococcus faecium*
XDR	Extensively drug-resistant bacteria
